# Modeling of miRNA and Drug Action in the EGFR Signaling Pathway

**DOI:** 10.1371/journal.pone.0030140

**Published:** 2012-01-11

**Authors:** Jian Li, Vikash Pandey, Thomas Kessler, Hans Lehrach, Christoph Wierling

**Affiliations:** Department Vertebrate Genomics, Max Planck Institute for Molecular Genetics, Berlin, Germany; Semmelweis University, Hungary

## Abstract

MicroRNAs have gained significant interest due to their widespread occurrence and diverse functions as regulatory molecules, which are essential for cell division, growth, development and apoptosis in eukaryotes. The epidermal growth factor receptor (EGFR) signaling pathway is one of the best investigated cellular signaling pathways regulating important cellular processes and its deregulation is associated with severe diseases, such as cancer. In this study, we introduce a systems biological model of the EGFR signaling pathway integrating validated miRNA-target information according to diverse studies, in order to demonstrate essential roles of miRNA within this pathway. The model consists of 1241 reactions and contains 241 miRNAs. We analyze the impact of 100 specific miRNA inhibitors (anit-miRNAs) on this pathway and propose that the embedded miRNA-network can help to identify new drug targets of the EGFR signaling pathway and thereby support the development of new therapeutic strategies against cancer.

## Introduction

MicroRNAs (miRNAs) are evolutionary conserved, endogenous, non-protein-coding, 18–22 nucleotide RNAs that exert function to negatively regulate gene expression at the post-transcriptional level in a sequence-specific manner [1]–[3]. miRNAs play important roles in nearly all biological processes, such as developmental timing, cell proliferation, apoptosis, stem cell maintenance, differentiation, signaling pathways, and pathogenesis including carcinogenesis [4]–[9]. To date, the human genome is predicted to encode approximately 1,000 miRNAs, equivalent to about 3% of the total number of human genes [10]. miRNAs negatively regulate target gene expression via complementary base pairing between their 5′ seed sequence and the target mRNA 3′ untranslated region. The 5′ “seed” region of the miRNA sequence (bases two to eight) is essential in mRNA target recognition [11]. miRNAs that bind to a protein encoding mRNA with imperfect complementarity repress the mRNA translation, whereas miRNAs binding to the mRNA with perfect complementarity target it for destruction [12]. The expression of approximately 30% of human proteins appears to be regulated by miRNAs [13]. Due to the relatively few complementary base pairs, the target spectrum of miRNAs can be very promiscuous. Although we do not know the precise number of targets of each miRNA, it is reasonable to suggest that the number could be in the hundreds. This means that a single miRNA can target multiple components of a single cellular pathway, or components of multiple pathways and therefore exert profound impact on cell biology [14]. This could put an individual miRNA in the unique position to function as a signaling amplifier, to convey signaling crosstalk between pathways or to confer signaling robustness of signaling pathways.

Cancer is a consequence of disordered genome function. A key challenge in cancer research is explaining how cancer cells acquire the fate of uncontrolled cell growth, aggressive invasion and destruction of adjacent tissue and at the same time ignore and circumvent apoptosis [15]. One approach in tumor therapy is to specifically manipulate deregulated intracellular signaling pathways and to reduce the aberrant signal produced by over-expressed oncogenes or alternatively to forcefully increase the expression of tumor-suppressor genes. A yet untested alternative approach might involve the use of miRNA targeting drugs that could benefit from the potential broad impact which a particular miRNA might have on multiple components within the same deregulated signaling pathway.

The EGFR signaling pathway is one of the most important cellular signaling pathways, which regulates relevant cellular processes, including proliferation, differentiation, and development [16]. Up-regulation and/or over-expression of EGFR signaling have been associated with cancer-related processes, including uncontrolled cellular proliferation and autocrine stimulation of tumors producing their own growth factors. EGFR also appears to protect cancer cells from toxic actions of chemotherapy and radiotherapy, rendering these treatment modalities less effective [17]–[19]. EGFR over-expression is frequently found in epithelial tumor entities such as gastric, colorectal, head-and-neck, breast, and lung cancers and is associated with advanced disease and poor clinical prognosis [20], [21].

In this study, we constructed a systems biological model of the EGFR signaling pathway including corresponding miRNA-target information based on diverse published miRNA studies (Table S1). We then conducted *in silico* analysis of the impact of miRNAs and their corresponding inhibitors on the EGFR signaling pathway, demonstrating the impact of miRNA regulatory processes on the behavior of this signaling pathway. Furthermore, we quantitatively elucidate the therapeutic concept “One hit – multiple targets” suggested by Wurdinger and Costa [22] hoping to open a new avenue for drug development in cancer research.

## Results

### 1. Establishment of an integrated miRNA-EGFR signaling pathway model

The miRNA-EGFR signaling model was constructed using the new version of PyBioS, a web-based modeling and simulation software [23], [24] (http://pybios.molgen.mpg.de). The model is based on molecular interactions and comprises 1241 reactions and 901 entities. The entities defined in the model are summarized in Table 1. The miRNA information is derived from miRBase [25] as well as extensive literature search. Among the implemented components, there are 26 sets of genes, compiled from groups of individual genes that have been assigned to similar biological functions. For instance, we defined MEK as a set of genes that includes the individual genes for MAP2K1 and MAP2K2, two closely related mitogen activated protein kinases. Furthermore, we implemented sets of miRNA genes whose mature miRNAs have the same targets in our model. For example, mir-631, mir-608, mir-604, mir-492, and mir-30a have the common targets TARBP2, RNASEN, DICER1 and DGCR8 [26]–[28]. Therefore, we defined a gene set entity, named mir-TRDD including these 5 miRNA genes. Similarly, there are mRNA sets, miRNA sets and protein sets which are produced by mRNA-transcription, miRNA-transcription and mRNA-translation processes, respectively.

**Table 1:** Summary of model components/reactions.

Component	Number	Reaction	Number
gene	183	transcription	179
mRNA	78	translation	75
protein	133	decay	218
miRNA	241	complex-formation	115
compound	35	translocation	133
complex	131	phosphorylation	49
pseudo-object	100	dephosphorylation	40
		activation	10
		miRNA-binding	417
		other types	5
Sum:	901	Sum:	1241

Compound is metabolite; complex includes protein-protein complex, protein-gene-complex, mRNA-miRNA complex; pseudo-object includes protein inhibitor and miRNA inhibitor.

**Figure 1:**
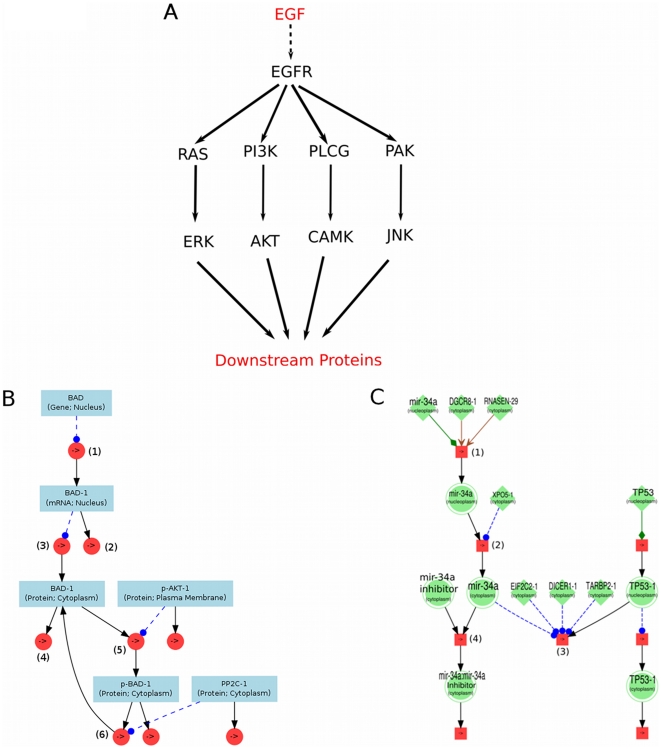
Overview of the model. **A**: The model of the EGFR signaling pathway contains 4 sub-pathways: MAPK pathway, PI3K-AKT pathway, Ca^2+^ signaling pathway, PAK signaling pathway. **B, C** are parts of the model network visualized by PyBioS. **B**: The mRNA BAD-1 is produced by the transcription reaction (1) of gene BAD and also takes part in decay reaction (2) and translation-reaction (3), which produces the protein BAD-1 (cytoplasm). The protein BAD-1 takes part in further three reactions that are the decay-reaction (4), phosphorylation reaction (5) and dephosphorylation reaction (6). The phosphorylated protein P-AKT (plasma membrane) catalyzes the phosphorylation reaction, in which protein BAD-1 is phosphorylated into P-BAD-1. Afterwards, the protein P-BAD-1 is then dephosphorylated; **C**: shows a simplified miRNA biogenesis, target recognition and competitive anti-miRNA effect. (1) miRNA-gene transcription; (2) miRNA translocation (from nucleus into cytoplasm); (3) miRNA-binding-target reaction; (4) miRNA binds to the miRNA inhibitor.

During construction of the model, we emphasized the biological sense, meaning that each protein entity is produced in the compartment cytoplasm by a translation reaction of a respective mRNA entity, which is generated in the compartment nucleus by the transcription reaction of a gene entity (Fig. 1B). Each mRNA and protein entity participate in their own decay reaction. We assume that each gene has basal expression. Therefore, the concentration of an mRNA entity depends on the concentration of its gene entity and the kinetic parameters of the corresponding mRNA transcription and decay reaction, the concentration of a protein entity depends on the concentration of its corresponding mRNA entity and the kinetic parameters of reactions, in which this protein entity takes part. The reactions are summarized in Table 1. Transcriptions include gene transcription and miRNA gene transcription, where gene transcription is defined as a one step process assuming that basal transcription is generated by constitutive action of a single transcription factor. The miRNA gene transcription simplifies two processes: (i) miRNA gene transcription catalyzed by DNA-polymerase II or DNA-polymerase III; (ii) cropping of the primary transcript (pri-miRNA) into a hairpin intermediate (pre-miRNA) by the nuclear 650 kDa microprocessor complex, comprising in humans of the RNase III DROSHA (RNASEN) and the DiGeorge syndrome critical region gene 8 (DGCR8) (see Fig. 1C). The miRNA binding target reaction simplifies two processes: (i) mature miRNA in complex with DICER and TARB binds to the Ago-complex and turns it into the RNA-induced silencing complex (RISC); (ii) RISC recognizes the target mRNA and binds to it (see Fig. 1C). Furthermore, we modeled clusters of distinct reactions involved in specific functions, such as EGFR, small GTPase signaling, MAPK cascade, phosphatidylinositol signaling, and Ca^2+^ signaling. Fig. 1A depicts the general overview of the EGFR signaling pathway of our model. The detailed model information is available under http://www.molgen.mpg.de/~sysbio/models/EGF_miRNA_model.html in the form of Systems Biology Markup Language (SBML) [29].

### 2. Analysis of predictive ability of the model

**Figure 2:**
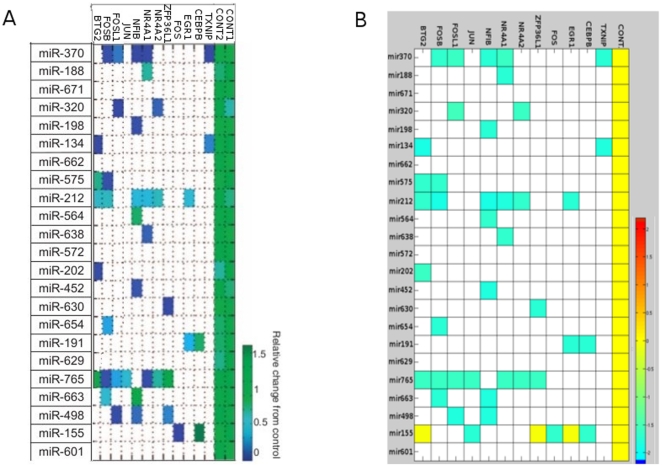
Comparison of model predictions with experimental results. **A**: Experimental results of relative concentration changes of target mRNAs according to individual miRNA over-expression experiments from the Avraham's study [30] (Reprinted with permission from AAAS); **B**: *in silico* prediction result of relative concentration changes of target-mRNAs according to each miRNA over-expression in the EGFR model. Both heatmaps show very similar qualitative results (protein down-regulation), the only discrepancies are for miR-155 and miR-498. mRNAs with low concentration changes (log2-ratio<0.001) are ignored and shown in ‘white’.

After establishing the miRNA-EGF signaling model we set out to validate its predictive value. Based on the simulation, we attempt to predict the dynamics of the underlying biological system so that the validity of the assumption can be tested. Therefore, the *in silico* prediction should be first compared with the experiment. If any inconsistency occurs at this stage, it indicates that the model representation is incomplete or not good enough. If the model could pass the initial validation, it can then be used to make predictions to be tested by experiments, as well as to explore many interesting questions that are not amenable to experimental inquiry. Hence, we compared the predictive results generated by our model with experimental data obtained in cell culture experiments. In a recent study, Avraham et al [30] investigated the impact of 23 miRNAs on the EGFR signaling pathway and used expression of multiple immediate early genes (IEGs) in EGF treated MCF10A cells as a transcriptional readout. In these cells, the authors individually over-expressed 23 miRNAs and then measured the concentration of IEGs mRNAs (Fig. 2A). We tested how well our model can reflect the observations of Avraham's study. Therefore, using Petri net simulations of our model (see Material and Methods), we simulated the effects of individual miRNA over-expression testing the same miRNAs that were analyzed in the aforementioned study (Fig. 2B). Notably, the model construction was performed independently from the Avraham's study, and we did not use any data from this study to feed into our model. The visual comparison of both results shows that our *in silico* simulation result is in good accordance with the *in vitro* response of MCF10A cells to miRNA over-expression (Fig. 2A,B). Therefore, the information on miRNA-target relationships we obtained by literature mining to build the model proves sufficient to qualitatively recapitulate the IEG transcriptional response to a cells miRNA expression status in the context of an activated EGFR pathway.

### 3. Simulation of putatively oncogenic properties of mir-192

Next we wanted to examine the inhibitory effect of a given miRNA on its putative targets within the EGFR signaling cascade. Based on the evidence provided by various studies, mir-192 regulates the expression of the genes MDM2 [31], EGFR [32], PIK3CA [32], TP53 [31], [33], PTEN [34], and CDKN1 [31]. We therefore chose to test the impact of mir-192 levels on the EGFR signaling response and performed simulations by varying mir-192 concentrations between 0 and 10,000 nM. We used this concentration range as expression of an individual miRNA is considered to vary widely in copy number per cell, with a few tissue-specific species up to more than 10,000 copies per cell (>2,000 nM) [35]. In these simulation experiments, expression of other miRNAs was omitted to examine the effect of individual miRNA regulation. Furthermore, the number of copies of an individual mRNA present in a single cell is considered to vary over four orders of magnitude (1 to >1,000 copies that is equivalent to 0.001 to 10 nM), with most mRNA species present in <100 copies and a few exceeding 1,000 copies [36]. Setting the concentration of ligand protein EGF to 1 nM should realistically provide a long and strong enough signal for its transduction through the entire signaling pathway during the course of simulation. Some compounds such as ATP and ADP were fixed to 1 nM, as well. Thereby, it is ensured that signal transduction will not halt only because of shortage of certain metabolites. The initial concentrations of other entities including mRNAs, miRNAs, proteins, complexes, etc. were set to 0 nM, which ensured the entire signaling pathway would only be affected by the EGF signal during simulation.

**Figure 3:**
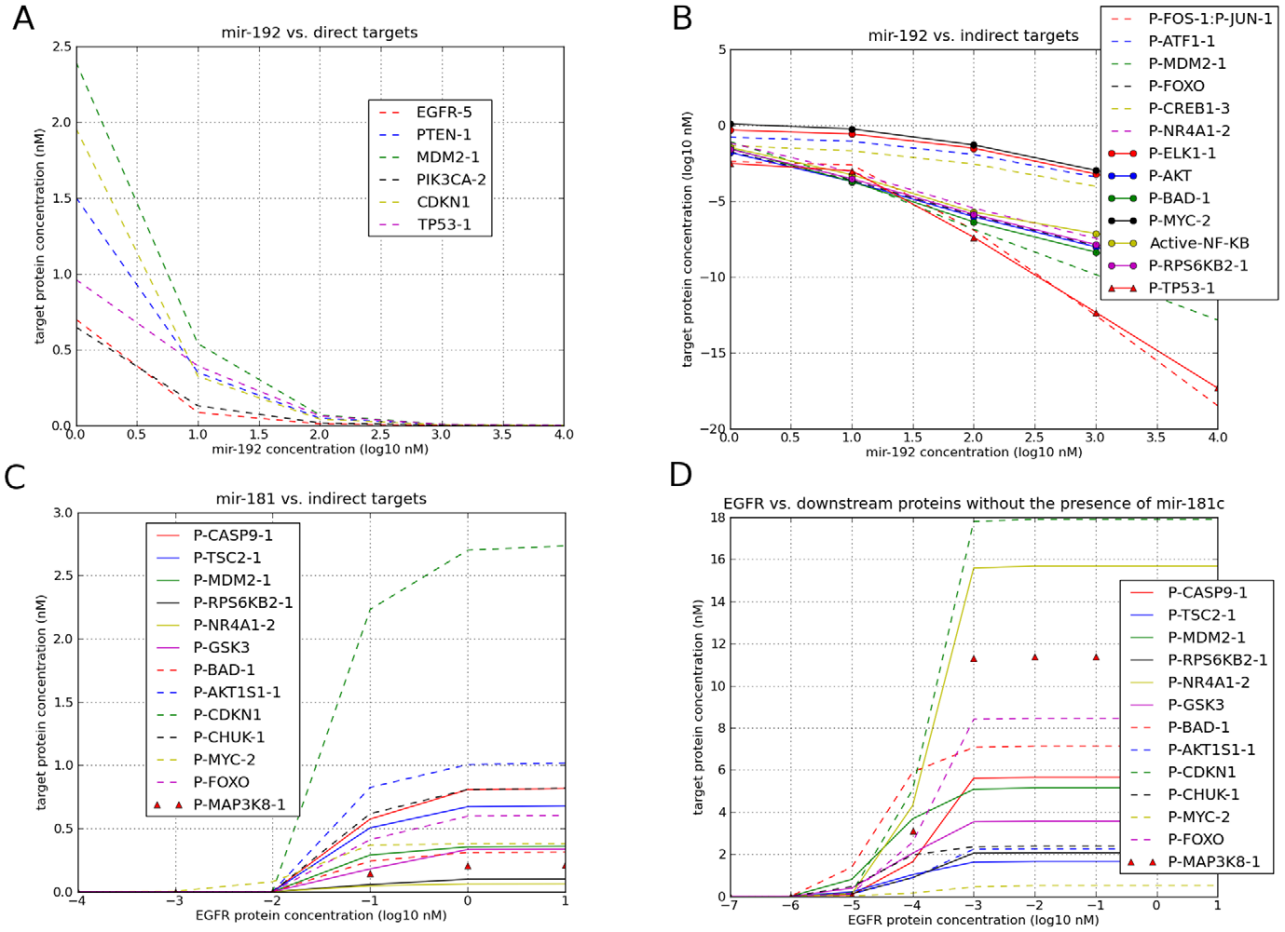
Modeling of mir-192 and mir-181c effects on the EGFR signaling pathway. **A**: Increased expression of mir-192 gene corresponds with a reduced level of targets' protein expression; **B**: Concentrations of EGFR downstream activated proteins are inversely correlated with the mir-192 gene expression level; **C**: simulation result with fixed mir-181c gene expression level (1 nM), whereas all other 240 miRNA genes are not expressed. All 13 AKT-dependent proteins can only be activated after the concentration of EGFR protein is getting higher than 10 pM. This activation threshold is due to the presence of mir-181c. **D**: represents another simulation result without the presence of mir-181c, additionally all other 240 miRNAs were not expressed, as well. At this condition, the activation threshold of these proteins is at 0.001 pM. By comparing these two results (**C**, **D**), one can understand that mir-181c raises the activation threshold of the EGFR signaling pathway significantly.

Modeling mir-192 over-expression using these experimental conditions reveals that the concentrations of mir-192 target proteins TP53, PTEN, MDM2 and CDKN1, are inversely correlated with the mir-192 gene expression levels (Fig. 3A). Interestingly, upregulated mir-192 gene expression is often seen in cancers [33], and based on our modeling results we suggest that mir-192 over-expression might confer a proliferative advantage of cancer cells by simultaneous suppression of the proteins TP53, PTEN, MDM2 and CDKN1. Notably, among the 241 miRNAs in our model, 44 miRNAs target TP53, 57 miRNAs target PTEN, and 10 target MDM2 and mir-192 is one of few miRNAs in our model that simultaneously target these 3 tumor suppressor proteins.

There is evidence that miRNA expression levels are correlated with the development of various human cancers suggesting that deregulated miRNAs might function as classical tumor suppressors and oncogenes [5], [37]. For example, mir-15a, mir-16 and let-7 have been found to be down-regulated in different types of cancer, suggesting that they can act as tumor suppressor genes [5]. The simulation result (Fig. 3A) indicates that mir-192 might function as an Oncomir by repressing the expression of several tumor suppressor genes simultaneously, which in turn reveals the important role of miRNAs within signaling cascades [2]. Since the expression of the EGFR protein is down-regulated by increasing mir-192 gene expression (Fig. 3A), the expression of most downstream proteins within the EGFR signaling pathway decreases (Fig. 3B) which illustrates the impact of a given miRNA on a signaling pathway by directly targeting its receptor protein. These results suggest that the oncogenic function of mir-192 might be partially compensated by reduced EGF signaling activity due to reduced EGFR protein levels. However, constitutive activating mutations in signaling components downstream of EGFR, like NRAS(Q61L) or BRAF(V600E), are common in cancer cells [38]. These mutations in combination with mir-192 over-expression could probably circumvent the effect of negative regulation of EGFR by mir-192 while retaining the putative mir-192 Oncomir effect via TP53, PTEN, MDM2 and CDKN1. Furthermore, it is noteworthy that there are 36 miRNAs in our model that target the EGFR mRNA (Table S2). It will certainly be interesting to analyze expression of these miRNAs in different cancer types with respect to the mutational status of the individual cancer case.

### 4. Simulation of a mir-181 dependent EGFR signaling threshold

AKT/PKB, apart from the MEK/ERK branch, is another important kinase branch activated downstream of mitogen-activated PI3K: EGFR+EGF→PI3K→PIP3→PDPK1→AKT→target proteins. According to previous studies, mir-181c targets AKT [39] and MYC [40]. Next, we were interested in how the EGFR signaling threshold (the minimal concentration of EGFR receptor protein for activating downstream proteins by constitutive input of the EGF signal) can be affected by mir-181c. Therefore, we set the initial condition of mir-181c gene expression level to 1 nM and the concentration of EGF fixed to 1 nM. During the simulation, the gene expression level of EGFR increases from 0.1 pM to 10 nM (Fig. 3C). At a threshold of 10 pM of EGFR, EGF can activate the downstream signaling proteins as judged by increasing concentrations of the chosen readout model components. In a second simulation, we simulated knock down of mir-181c by setting its gene expression level to zero, while all other conditions remained identical (Fig. 3D). In the absence of mir-181c, the threshold of the EGFR signaling activation is lowered to 0.001 pM, which indicates that the presence of mir-181c can potentially contribute to fine tuning of the EGFR signaling threshold in a range from 0.001 pM to 10 pM of the EGFR protein under these simulation conditions. Our results therefore exemplify a crucial role that miRNAs might play in signaling cascades by dampening positive mediators, restricting the signal to an appropriate competence zone. Hence, miRNAs targeting important components of a signaling pathway might be able to regulate their target to achieve optimal signaling efficacy and limit undesired signaling fluctuations, which is essential for genes expressed at low levels, because stochastic changes in the activation of their promoter are more genetically prominent [41].

### 5. Evaluation of the “One hit – Multiple Targets” concept using miRNA modeling

Evidence from various studies indicates that normal miRNA expression is important for proper development and differentiation in a tissue and cell-type specific manner [22]. Deregulation of miRNA expression is therefore a common feature of many types of cancer [42]. However, one important question remains untouched: whether the miRNAs are differentially expressed as a consequence of the pathologic cell state, or whether the particular cancer or disease is a direct cause of the deregulated expression of miRNAs. However, the therapeutic concept “One Hit – Multiple Targets” could provide a solution to this problem.

**Figure 4:**
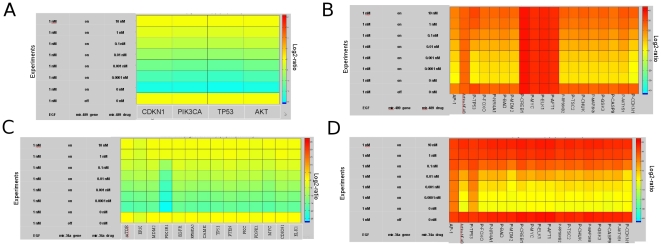
Modeling of anti-mir-489 and anti-mir-34a effects on the EGFR signaling pathway. Heatmaps **A** and **B** are the results of anti-mir-489 simulations. **C** and **D** are the results of anti-mir-34a simulations. The first experiment within each heatmap (from bottom to top) is the ‘control’ state; the miRNA effect and anti-miRNA effect of the different experiments is always compared versus the ‘control’ state. **A**: Quantitative changes of mir-489 direct target-proteins due to different amounts of anti-mir-489 per experiment. The inhibition effects of mir-489 is inversely correlated with the anti-mir-489 concentration. **B**: Quantitative changes of the EGFR downstream activated proteins according to different amounts of anti-mir-489 per experiment. The concentrations of many downstream activated proteins of the EGFR signaling pathway correlate with the concentration of anti-mir-489. **C**: Quantitative changes of mir-34a direct target-proteins due to different amounts of anti-mir-34a per experiment. The inhibition effects of mir-34a inversely correlate with the anti-mir-34a concentration. **D**: Quantitative changes of the EGFR downstream activated proteins according to different amounts of anti-mir-34a per experiment. The concentrations of many downstream activated proteins correlate with the anti-mir-34a concentration.

The idea behind this concept from Wurdinger's study [22] is to normalize or to correct a deregulated miRNA expression, which then directly or indirectly affects its protein-coding mRNA targets. Some of these targets may be encoded by oncogenes and tumor suppressor genes and the defects in those mRNA expression levels could be immediately reverted to normal state by normalization of deregulated miRNA expression. This restoration of the deregulated post-transcriptional control could have immense therapeutic benefit. In the following, we perform *in silico* simulation to quantitatively elucidate the effect of two miRNA inhibitors on the EGFR signaling pathway: anti-mir-489 and anti-mir-34a. In general, anti-miRNAs could be designed in form of antagomirs, which are anti-miRNA oligonucleotides (AMOs) conjugating with cholesterol [43]; alternatively, they can be designed as locked-nucleic-acid anti-sense oligonucleotides (LNAs) [44], [45]. In both cases, specific anti-miRNAs bind to their corresponding mature endogenous miRNA according to sequence complementarity, thus effectively blocking miRNA inhibitory function. The anti-mir-489 specifically antagonizes the inhibition effect of mir-489, which targets CDKN1, PIK3CA, TP53 and AKT [46], and anti-mir-34a antagonizes the inhibition effect of mir-34a, which targets MTOR, ERK, MDM2, PIK3R1, EGFR, RPS6KA5, CAMK, TP53, PTEN, PKC, PDPK1, MYC, CDKN1 and ELK1 [47]–[54]. In order to study the potential anti-miRNA effects *in silico*, we carried out two simulation processes and separately investigated both inhibitor effects and additionally the downstream effect on the EGFR signaling pathway. Therefore we performed *in silico* experiments with different concentrations of anti-mir-489 (Fig. 4A and B). The heatmap of Fig. 4A shows the change in concentration of mir-489 target proteins, whereas Fig. 4B depicts the change of concentration of the downstream proteins in the EGFR signaling pathway. The results show a correlation between the concentrations of these four target proteins and the amount of anti-mir-489, which indicates that the miRNA-489 inhibitory effect is effectively abolished by this anti-miRNA. Actually, anti-mir-489 exerts its inhibitor effect not only on these four target proteins of mir-489, but also indirectly influences downstream proteins of the EGFR signaling pathway through those target proteins (Fig. 4B). This reflects the “One Hit – Multiple Targets” effect of anti-miRNA. According to the simulation results (Fig. 4.), we expect a functional relevant concentration range of an anti-miRNA drug to be in between 10 nM and 100 nM, which suggests a suitable range to test the potential drug effect in, for example, cultured cancer cell lines.

We also performed *in silico* experiments with different concentrations of anti-mir-34a (Fig. 4C and 4D). The anti-mir-34a inhibits mir-34a in the same way as anti-mir-489 inhibits mir-489. The common phenomenon of the application of both anti-miRNAs is that not only the expression levels of target proteins (Fig. 4 A and C), but also the indirect target proteins (Fig. 4B and D) can be reverted to the normal expression levels (the “control” state), by increasing anti-miRNA concentrations to a certain level (10 nM). Both results reveal the crucial role of “One Hit – Multiple Targets” in the signaling cascade. ‘Correcting’ miRNA inhibitory effect can lead to normalizing the expression levels of its numerous target proteins, some of which may function as onco-proteins and tumor-suppressor proteins. This might result in recovery of the normal phenotype of a cell from a disease state to a normal state. However, there are also differences between the effect of mir-34a and anti-mir-489, for instance, there are five proteins (Active-NF-kB, P-CREB1, P-MYC, P-ELK1 and P-AFT1), which do not change at all due to anit-mir-489 (Fig. 4B), while all the downstream proteins were affected by mir-34a and its anti-mir (Fig. 4D). It seems that anti-mir-34a has more profound effects on this signaling pathway than anti-mir-489 does. However, this could be due to the fact that mir-34a targets 14 proteins in the EGFR signaling pathway, while mir-489 targets only four.

**Figure 5:**
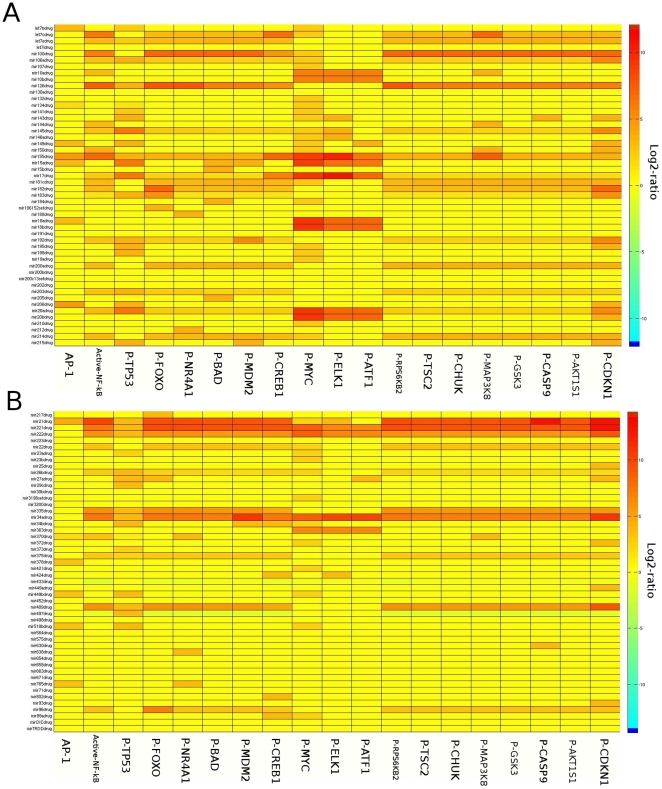
Modeling the individual effect of 100 anti-miRNAs. **A and B**: Each anti-miRNA is activated when the corresponding miRNA is over-expressed individually (an anti-mir-combination effect is omitted). Each row in this heatmap represents the predicted anti-miRNA effect and the more columns in the same row appears red or orange, the stronger is the predicted effect that this particular miRNA inhibitor can exert on the EGFR signaling pathway. In this manner, we can examine the impact of each anti-miRNA on this signaling pathway.

Next, we assessed the potential effects of 100 different anti-miRNAs individually and selected 19 important downstream proteins of the EGFR signaling pathway as readout components of effectiveness of anti-miRNAs. An anti-mir-combination effect was not considered. Using this *in silico* simulation approach, we found clear differences of anti-mir effects on this signaling pathway. For instance, anti-mir-155 has considerable influence on the activation of many downstream proteins and is therefore considered as an effective anti-miRNA (Fig. 5A), whereas anti-mir-663 exerts almost no effect on this pathway and is therefore regarded as a non-effective or less effective anti-miRNA for this signaling pathway (Fig. 5B). Furthermore, based on this simulation data, we investigated the concentration changes of all model components due to the effect of each anti-miRNA, applied a statistical t-test and calculated the P-value of each anti-miRNA according to its effect on the entire signaling pathway (see Materials and methods). Table 2 lists the top 15 anti-miRNAs with most significant P-values (P-value<0.05). Thus, we believe that those anti-miRNAs should be suitable as potential candidates for cancer treatment. However, those anti-miRNAs need to be tested and verified by *in vitro* and *in vivo* experiments.

**Table 2:** Top 15 anti-miRNAs.

anti-miRNA	P-value
Anti-mir-21	1.90e-06
Anti-mir-155	4.54e-06
Anti-mir-221	3.64e-04
Anti-mir-17	1.44e-03
Anti-mir-489	1.78e-03
Anti-mir-222	1.81e-03
Anti-mir-335	1.85e-03
Anti-mir-126	1.86e-03
Anti-mir-100	3.50e-03
Anti-mir-181c	1.17e-02
Anti-mir-214	1.18e-02
Anti-mir-200a	1.19e-02
Anti-mir-34a	1.26e-02
Anti-let-7c	3.22e-02
Anti-mir-182	4.22e-02

P-value gives the significant level of concentration-differences of all model components between the state of the applied specific anti-miRNA and control state (inactivated anti-miRNA).

**Figure 6:**
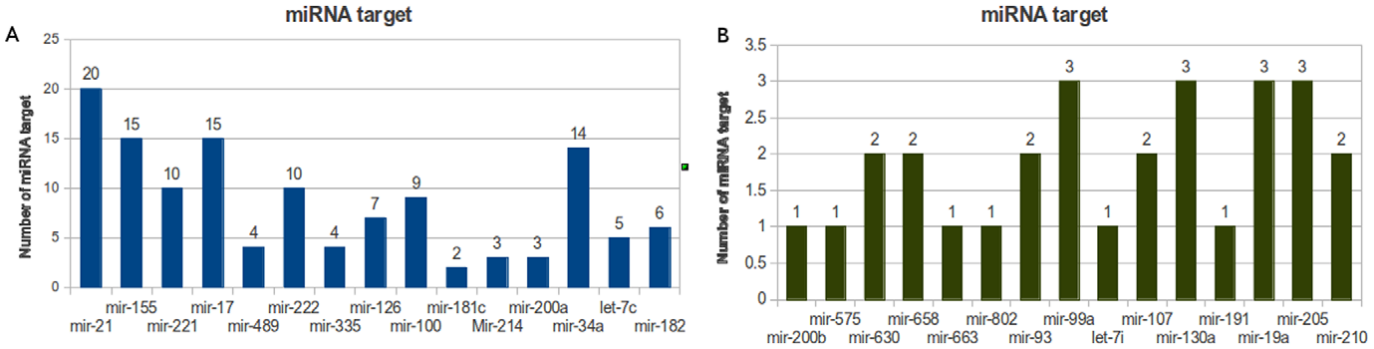
Histogram of miRNA/target relationships. **A**: The top ranked 15 miRNAs of Table 2 correlated with the amount of their corresponding targets in the EGFR signaling pathway (Table 1). **B**: The 15 miRNAs correlated with amount of their corresponding targets in EGFR signaling pathway. The anti-miRNAs of these miRNAs have much less effect on this pathway (Fig. 5A and B).

Afterwards, we investigated the corresponding 15 miRNAs with their target information and another 15 miRNAs whose anti-miRNAs have almost no effect on the EGFR signaling pathway (Fig. 6A and B). We counted the number of targets of those miRNAs individually according to the information in Table S1. As expected, we found that an anti-miRNA that antagonizes a miRNA with more targets, can exert a greater impact on the signaling pathway. In contrast, an anti-miRNA appears less effective, if the anti-miRNA antagonizes a miRNA with very few targets. However, anti-mir-181c, anti-mir-214 and anti-mir-200a can also exert considerable impact on this signaling pathway during simulation, although the number of direct targets of these three miRNAs are low (Fig. 6A). The reason for this could be that they target key components of the EGFR signaling pathway, for instance, mir-181c targets AKT and MYC (see above). This phenomenon helps to understand the principle of “One Hit – Multiple Targets” approach.

## Discussion

Since the discovery of miRNAs in 1998, the function of those molecules in oncogenesis and cancer progression has been a major subject of intense investigation. One way to conceptualize the roles of the miRNAs is in the context of an integrated network emerging from summation of the interactions of miRNAs and their targets. In this study, we constructed a systems biological miRNA-model of the EGFR signaling pathway using the software PyBioS. The miRNA-target information is based on diverse studies (Table S1). The absorption of an extracellular signal, in this case EGF ligand, must not induce some specific cellular instructions. Actually, it is the cell that interprets the signal according to its history and actual environment. In this case, our model can be considered as a virtual cell functioning only with the EGFR signaling pathway and the initial conditions of the model can be viewed as the virtual environment in which this virtual cell is living. We showed the predictive ability of our model by comparing the *in silico* simulation data with the experimental results from the study of Avraham et al [29] and reached high accuracy. Therefore, we conclude that our model should be able to predict cellular responses to altered miRNA levels. Based on this model, we conducted quantitative miRNA and anti-miRNA *in silico* analysis of the effects of miRNA expression levels and the resulting alterations evoked by miRNA inhibitors. We demonstrated that some miRNAs could play an essential role in the EGF triggered signaling cascade and some anti-miRNAs might have considerable effect on this pathway. For instance, by increasing the expression level of mir-192 in our model, we showed that not only the expression of its direct target proteins were reduced, but also many downstream proteins were affected indirectly, indicating that altered miRNA expression level might have a direct and indirect impact on the EGFR signaling pathway. Furthermore, we demonstrated that mir-181c is able to raise the activation threshold of the EGFR signaling pathway, which potentially optimizes the signal efficacy, and limits undesired signaling fluctuations. We demonstrated that by administration of a proper amount of anti-mir-34a and anti-mir-489 *in silico*, the direct and indirect target proteins of the EGFR signaling pathway could be reverted to a normal state, which indicates that a phenotype of a cell from a disease state could be reverted to a normal state by anti-miRNAs inhibition and elucidates the therapeutic benefit of anti-miRNA drugs.

Moreover, we studied 100 different anti-miRNAs by investigating their effect on the downstream proteins of the EGFR signaling pathway. As expected, our simulation results indicate that the more targets a miRNA has in the EGFR signaling pathway, the more likely is the corresponding anti-miRNA to exert considerable effect on this pathway. However, we show that targeting key components of the signaling pathway is more important and effective. Based on the simulation data, we propose a top 15 list of anti-miRNAs (p<0.05) with most significant impact on the EGFR signaling pathway (Table 2), and suggest that those anti-miRNAs could be potential test candidates for follow-up studies assessing the impact of distinct miRNA inhibitors on EGFR pathway activation in cancer derived cell lines or other suitable model systems. One particularly interesting candidate could be anti-mir-21. mir-21 has been implicated as an oncogene in different types of cancers such as breast-, colon-, cervical-cancer and glioblastoma and a higher level of mir-21 is often correlated with poorer survival of patients [55], [56], [57]. Based on our simulation results, the inhibition of mir-21 leads to a significant up-regulation of pro-apoptotic proteins such as CASP9, BAD, TP53. This is in agreement with the study conducted by Chan et al [55], where mir-21 has been shown to be an anti-apoptotic factor. Moreover, the inhibition of mir-21 also leads to the up-regulation of CDKN1, a tumor-suppressor that regulates the cell cycle progress [58], [59]. The down-regulation of CDKN1 supports the oncogenic activity of mir-21. In 2010, an independent study of Mei et al [60] successfully applied an anti-mir-21 to enhance the chemotherapeutic effect in breast carcinoma cells. Based on our simulation results, we suggest that an anti-mir-21 (p-value: 1.90e-06) could be an effective anti-cancer drug also for other types of cancer with elevated EGFR signaling. Furthermore, according to our simulation results, the application of anti-mir-335 can lead to the up-regulation of many essential tumor suppressors such as CDKN1, TP53, CASP9 and TSC2. In 2011, an independent study of Shu et al [61] elucidated the oncogenic potential of mir-335 and validated that the inhibition of mir-335 can inhibit the growth and invasion of malignant astrocytoma cells. Therefore, we suggest that anti-mir-335 (p-value: 1.85e-03) might be an effective anti-cancer drug for other types of cancer, similar to the potential benefits of an anti-mir-21. Similarly, the anti-mir-221 (p-value: 3.64e-04) and anti-mir-222 (p-value: 1.81e-03) exerted significant impact on model components during in silico simulation. In 2011, Stinson et al [62] substantiated that specific miRNA such as mir-221 and mir-222 can promote transformation to more aggressive cancer phenotype with poor diagnosis. Hence, the therapeutic value of the application of both anti-mir-221/222 should not be underestimated. We therefore conclude that our modeling results with respect to the impact of miRNAs on the EGFR pathway are in good accordance with published data of some of the miRNAs that are relevant in cancer treatment. The other eleven anti-miRNAs we identified to have the greatest impact on the EGFR signaling pathway (Table 2) have not been tested for their anti-oncogenic properties in vivo and we suggest that they should be of importance for in vitro or *in vivo* follow-up studies.

Surprisingly, we noticed that among the 241 miRNAs of our model, 130 miRNAs target DICER, and 45 miRNAs target RNASEN (Table S2), whose protein products process pre-miRNA into mature, functional miRNA thereby playing an essential role in the miRNA-biogenesis process. This raises the interesting question whether those 175 miRNAs regulate the overall mature miRNAs level through targeting the mRNAs of both genes. William et al [63] discovered that in some types of tumor, such as ovarian cancer, a lower DICER expression was significantly associated with advanced tumor stage. DICER down-regulation has also been discovered in various cancer types by other groups [64]–[66]. This DICER misregulation could reflect a globally impaired expression of mature miRNAs in human cancers [67], [68].

One limitation of our current EGFR signaling model is that ErbB2–4 are not taken into consideration and we analyze EGFR (ErbB1) as a single and isolated cell surface receptor. The future work could be adding the combination of homo- and heterodimerization of these four ErbB family members. Furthermore, our model assumes that each gene has basal expression and we have not added specific transcription factors or expression data for all transcription reactions. Similarly, for the phosphorylation reactions, we have not considered the detailed information of the phosphorylation sites of proteins. Despite of those flaws, our model could effectively be employed for estimating miRNAs inhibition effect.

Recently, RNAi based methodologies have been widely used in order to silence a single target gene, and have contributed to advances in molecular biology [69], [70]. However, various studies using microarray based expression screens have indicated that in pathological tissues there can be an imbalanced gene expression pattern involving many genes [71], [72], which can reduce the usefulness of those RNAi technologies dramatically. In contrast, the concept that one miRNA can manipulate several genes involved in one or many signaling pathway seems to be very promising and effective for cancer treatment. Furthermore, Iguchi et al [57] pointed out that the therapeutic intervention of miRNA should less likely have severe adverse effect. However, the studies conducted by Okamura et al [73] and Calin et al [74] explain the passenger strand effect, meaning that the passenger strand of the mature miRNA exerts an inhibitory effect and confers resistance to miRNA regulation, respectively. These finding indicate that cancerous cells are able to eliminate the therapeutic effect of a miRNA. Therefore, we believe that a combined therapeutic strategies might be needed to succeed in clinical application of anti-miRNA drug.

## Materials and Methods

### 1. Petri Net Extension

The simulation processes are based on the Petri net, which is a graphical and mathematical modeling language developed in the early 1960s by Carl Adam Petri [75]. The Petri nets approach has subsequently been adapted and extended in many fields such as systems biology. Furthermore, many extensions to Petri nets have been developed for various modeling and simulation purposes [76]. We implemented a Petri nets extension for the PyBioS software [24], which includes the characteristics of hierarchical-, hybrid- and timed-Petri nets. Therefore, it is particularly suitable for the simulation of large-scale networks. In the following, we explain the simulation process.

**Figure 7:**
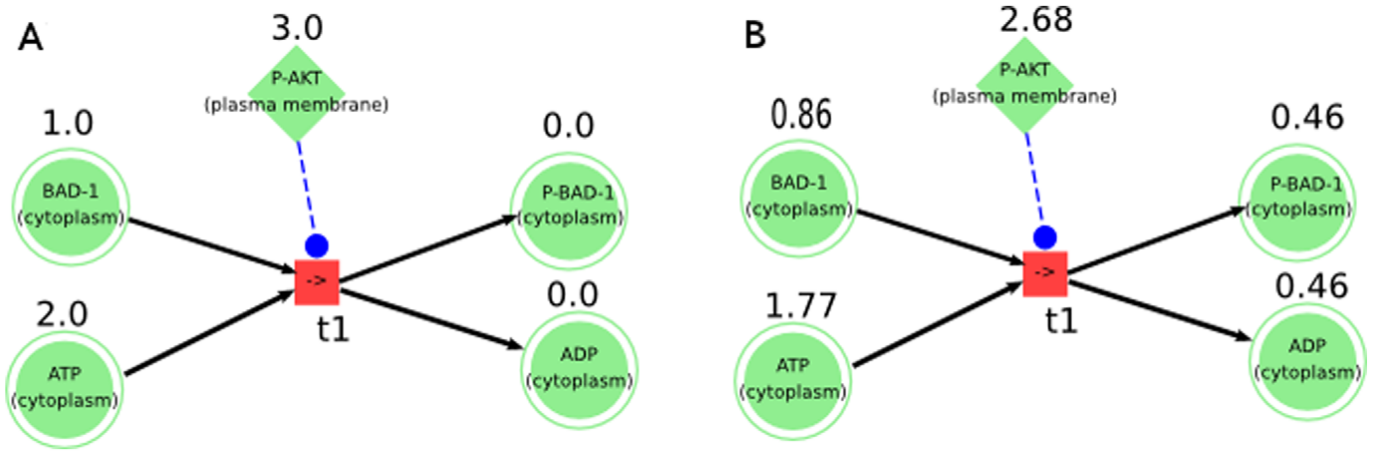
Transition fire of Petri Nets. An exemplary phosphorylation reaction of the Petri Nets. BAD-1 (p1), ATP (p2), P-BAD-1 (p3), ADP (p4) and P-AKT (p5) are places and phosphorylation reaction (t1) is a transition. **A**: the concentrations of model components at time point 0 (before transition fire); **B**: the concentrations of components in model at time point 1 (after transition fire).

In our model network, places and transitions represent bio-objects (gene, mRNA, miRNA, protein and compound) and biochemical reaction, respectively (Fig. 7). Each place is marked with a value representing the concentration of a model component.

Formal definition of Petri nets [77]:

A Petri net is a 6-tuple, PN = (P, T, F, W, m, D)

where:

P = { p_1_, p_2_ , … , p_x_}is a finite set of places (model contains x bio-objects),
T = { t_1_, t_2_ , … , t_y_} is a finite set of transitions (model contains y reactions),
F is subset of [( P x T ) U (T x P ) is a set of arcs]
W: F→{0, 1, 2, ‥ x } is a weight function,
m: P→{0, 1, 2, ‥ x } is the initial marking,
P ∩T = Ø (meaning that the sets P and T are disjointed),
D: m→P→{p_1_, p_2_ , … , p_x_} is the decay rate function of each component.

During simulation in each time step, each transition is evaluated with transition speed as ‘activated’ or ‘not activated’. If an activated transition can fit to a certain criteria, then it can fire, referring to biological sense, this reaction can occur. The transition speed is calculated by individual functions F_y_(S, E, I) referring the corresponding substrate S, enzyme E and Inhibitor I for each reaction y.

For instance, the speed of transition t1:
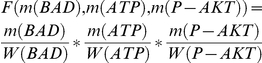
where m(BAD), m(ATP) and m(P-AKT) are concentrations of places BAD, ATP and P-AKT; W(BAD), W(ATP) and W(P-AKT) are the weights of the places.

If the following conditions hold true:
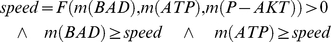
the transition t1 fires in forward direction, meaning that this phosphorylation reaction takes place.

If the following conditions hold true:
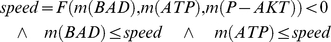
the transition t1 fires in backward direction, meaning that the dephosphorylation reaction takes place.

If one of the above two conditions is fulfilled, but the enzyme P-AKT is present at low concentration, the reaction can take place. However, its reaction flux will be low, too.

For example, at time point 0, initial concentrations of BAD-1, ATP and P-AKT are 1.0, 2.0 and 3.0, and we assume that their weight function values are
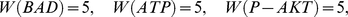
then the decay function values are
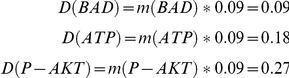
and the speed is calculated by f1 :
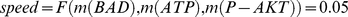

Table 3, 4 show the concentration-changes of components of reaction t1 from the time point 0 to time point 1.

**Table 3:** Transition t1 at time point 0 (before firing).

Components	Concentrations
BAD-1	1.0
ATP	2.0
P-AKT	3.0
P-BAD-1	0.0
ADP	0

**Table 4:** Transition t1 at time point 1 (after firing).

Components	Concentrations
BAD-1	0.95 – 0.09
ATP	1.95 – 0.18
P-AKT	2.95 – 0.27
P-BAD-1	0.05 – 0.05*0.09
ADP	0.05 – 0.05*0.09

It is noteworthy to mention that we take multiple time scales of intracellular biochemical reactions into consideration. For proteins and components, the decay takes place each time according to their individual decay function D(m(proteins)*0.09) with 0.09 as decay kinetic parameter; for ligand-receptor complex, the decay takes place every 10 times according to their individual D(m(ligand-receptor complex)*0.2) with 0.2 as decay kinetic parameter. Currently, these different decay kinetic parameters are based on empirical experience. All types of kinetic parameters applied in Petri Nets simulation are summarized in Table 5.

**Table 5:** The summary of kinetic parameters applied in the Petri nets simulation.

Reaction Type	Kinetic Parameter	Reaction Type	Kinetic Parameter
Complex Formation	0.55	Translocation	0.8
Phosphorylation	0.35	Translocation (mRNA)	0.5
Dephosphorylation	0.01	Dephosphorylation(enzym)	0.15
Transcritpion	0.5	Inhibitory parameter	1.5
Decay	0.02	other parameter	0.35

### 2. Analysis of significant changes of model components due to the effect of anti-miRNAs

Significance analysis of concentration changes of all model components were performed using t-test [78]. Suppose the model contains *n* components and C_X_ symbolizes the simulated concentration of one model component and for the state of the application of anti-miRNA (e.g. anti-mir-21)
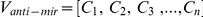
where C_x_ is the concentration of one model component in the current state, and for the corresponding control state (e.g. mir-21)
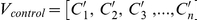
where C′_x_ is the concentration of one model component in the control state the p-value is copmuted by the t-test:
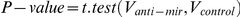



## Supporting Information

Table S1 Reference list of miRNA-targets.
(TXT)

Table S2 Classification of miRNAs according to their targets.
(TXT)
